# Identification of an Annonaceous Acetogenin Mimetic, AA005, as an AMPK Activator and Autophagy Inducer in Colon Cancer Cells

**DOI:** 10.1371/journal.pone.0047049

**Published:** 2012-10-08

**Authors:** Yong-Qiang Liu, Xin Cheng, Liang-Xia Guo, Chan Mao, Yi-Jie Chen, Hai-Xia Liu, Qi-Cai Xiao, Sheng Jiang, Zhu-Jun Yao, Guang-Biao Zhou

**Affiliations:** 1 Division of Molecular Carcinogenesis and Targeted Therapy for Cancer, State Key Laboratory of Biomembrane and Membrane Biotechnology, Institute of Zoology, Chinese Academy of Sciences, Beijing, China; 2 Graduate University of the Chinese Academy of Sciences, Beijing, China; 3 State Key Laboratory of Coordination Chemistry, Nanjing National Laboratory of Microstructures, School of Chemistry and Chemical Engineering, Nanjing University, Nanjing, China; 4 Guangzhou Institute of Biomedicine and Health, Chinese Academy of Sciences, Guangzhou, China; University of California Irvine, United States of America

## Abstract

Annonaceous acetogenins, a large family of naturally occurring polyketides isolated from various species of the plant genus *Annonaceae*, have been found to exhibit significant cytotoxicity against a variety of cancer cells. Previous studies showed that these compounds could act on the mitochondria complex-I and block the corresponding electron transport chain and terminate ATP production. However, more details of the mechanisms of action remain ambiguous. In this study we tested the effects of a set of mimetics of annonaceous acetogenin on some cancer cell lines, and report that among them AA005 exhibits the most potent antitumor activity. AA005 depletes ATP, activates AMP-activated protein kinase (AMPK) and inhibits mTOR complex 1 (mTORC1) signal pathway, leading to growth inhibition and autophagy of colon cancer cells. AMPK inhibitors compound C and inosine repress, while AMPK activator AICAR enhances, AA005-caused proliferation suppression and subsequent autophagy of colon cancer cells. AA005 enhances the ATP depletion and AMPK activation caused by 2-deoxyglucose, an inhibitor of mitochondrial respiration and glycolysis. AA005 also inhibits chemotherapeutic agent cisplatin-triggered up-regulation of mTOR and synergizes with this drug in suppression of proliferation and induction of apoptosis of colon cancer cells. These data indicate that AA005 is a new metabolic inhibitor which exhibits therapeutic potentials in colon cancer.

## Introduction

In tumor cells there is an increased glycolytic pathway enzymes and glucose transporters even in the presence of a high O_2_ concentration, ultimately leads to an elevated ATP production rate [1]. Clinical evidence has linked cell metabolism with cancer outcomes, and reprogramming energy metabolism has been approved to be an emerging hallmark of cancer [2]. These observations have raised interest in targeting energy metabolism for cancer therapy for both hypoxic (glycolytic) and oxidative tumors [1], albeit also concerns that these therapies would have unacceptable effects on normal cells. Intriguingly, some of the first cancer therapies targeting specific metabolic needs of cancer cells remain effective in the clinic today, re-inspiring efforts to target metabolic dependencies of cancer cells as a selective anticancer strategy [3].

AMP-activated protein kinase (AMPK) which exists in cells as a heterotrimeric complex composed of a catalytic kinase subunit (α) and two regulatory subunits (β and γ), is a sensor of energy status that maintains cellular energy homeostasis. It is activated by a fall in ATP (concomitant with a rise in ADP and AMP) or stimuli that increase the cellular AMP/ATP ratio, resulting in the activation of catabolic pathways and the inhibition of anabolic pathways [4], [5]. Essential to activation of AMPK is its phosphorylation at Thr-172 by an upstream AMPK kinases (AMPKKs) [6] and tumor suppressor LKB1 [7] which is a serine/threonine kinase associated with gastrointestinal polyposis and cancer [8] and lung cancer [9]. AMPK phosphorylates two rate-limiting enzymes in fatty acid and cholesterol synthesis: acetyl-CoA carboxylase (ACC) and HMG-CoA reductase, as well as other downstream targets, culminating in the inhibition of anabolic pathways and the activation of catabolic pathways [5]. AMPK activation directly limits translational initiation and protein synthesis [10], through inhibition of translation elongation factor 2 (EF2) [11], and indirectly through TSC2, leading to suppression of mammalian target of rapamycin (mTOR) [12] which can phosphorylate and activate p70 S6 kinase and 4E-binding protein (4EBP) [13].

AMPK activation by AMP analog 5-aminoimidazole-4-carboxamide ribonucleotide (AICAR) accumulates cyclin dependent kinase inhibitors p21 and p27 and down-regulates cyclin D1 in human hepatocellular carcinoma cells, leading to cell cycle arrest at G1 phase [14]. Population studies provide clues that the use of metformin which is an AMPK activator, may be associated with reduced incidence and improved prognosis of certain cancers [15], [16]. In breast cancer, metformin exerts inhibitory effects via inhibition of mTOR-dependent translation initiation [17], [18]. Metformin inhibits proliferation, decreases cell viability and blocks cell cycle in G1 phase in prostate cancer cells, and in vivo treatment with metformin leads to a significant reduction of tumor growth in mice bearing xenografts of prostate cancer cells [19]. Metformin and AICAR induce apoptosis and suppresses the tumor growth of colon cancer line HCT116 p53(−/−) xenografts, and trigger autophagy of HCT116 p53(+/+) cells [20]. AICAR and protein folding inhibitor 17-AAG, especially when combined, show efficacy against aneuploid human cancer cell lines [21]. These results indicate that AMPK could be a rational drug target and lead compounds should be identified or designed for the development of therapeutic avenues for cancers.

Annonaceous acetogenins represent a class of naturally occurring polyketides isolated from various species of the plant genus *Annonaceae* [22]. These compounds exhibit diverse bioactivities, including promising cytotoxicites and antiparasitic activities [23]. Though studies show that annonaceous acetogenins can disrupt mitochondrial function through blocking mitochondria complex I and ubiquinone-linked NADH oxidase [23], and bind the third matrix-side loop of ND1 subunit in mitochondrial NADH-ubiquinone oxidoreductase [23], [24], the mechanisms of action of these compounds in fighting cancer remain largely unknown. The chemistry group of our team had designed and synthesized a series of annonaceous acetogenin mimetics [25]–[28]. In this work we tested the biological activity of some new analogs and investigated the mechanisms underlying annonaceous acetogenins’ cytotoxicities, and reported that a mimetic AA005 which showed potent and selective inhibitory activities against a variety of cancer cells, was able to activate AMPK and induce cell cycle arrest followed by autophagy, demonstrating its therapeutic potentials.

## Materials and Methods

### Chemicals and Reagents

Annonaceous acetogenin mimetics (Table 1) were dissolved in DMSO and stored at −20°C. The 3-(4,5-Dimethylthiazol-2-yl)-2,5-diphenyltetrazolium bromide (MTT) was purchased from Amresco Inc. (Solon, OH). Compound C, rapamycin, rotenone, inosine, Rhodamine 123, 2-DG and AICAR were purchased from Sigma. MitoTracker Deep Red FM was purchased from Invitrogen. ATP Assay Kit was purchased from Beyotime Institute of Biotechnology (Haimen, Jiangsu, China).

**Table 1:** IC_50_ values of the derivatives of AA005 in various human cancer and noncancerous cell lines.

No.	Compound	Structure	MW	IC50 (µM)					
				MCF7	SGC7901	HCT116	HT29	BEAS-2B	HLF
1	AA005	<$>\scale 75%\raster="rg1"<$>	554.8	0.26±0.05	0.06±0.02	0.11±0.008	0.35±0.09	>50	20.9±7.4
2	AA090	<$>\scale 75%\raster="rg2"<$>	320.3	>50	>50	>50	>50	>50	>50
3	AA091	<$>\scale 75%\raster="rg3"<$>	498.4	4.4±0.042	1.5±0.3	4.3±0.47	19.5±3.15	>50	>50
4	AA093	<$>\scale 75%\raster="rg4"<$>	570.45	0.18±0.05	0.03±0.01	0.13±0.01	0.38±0.1	31.2±5.18	18.3±2.78
5	AA101	<$>\scale 75%\raster="rg5"<$>	678.5	5.95±0.59	2±0.39	5.18±0.18	>50	>50	>50
6	AA102	<$>\scale 75%\raster="rg6"<$>	766.6	1.8±0.18	0.44±0.01	2.3±0.16	9.9±1.47	>50	5.8±0.62
7	AA103	<$>\scale 75%\raster="rg7"<$>	723.0	1.9±0.66	0.27±0.03	1.1±0.08	3.3±0.97	9.3±0.98	4.9±0.17
8	AA104	<$>\scale 75%\raster="rg8"<$>	811.1	2.5±0.59	0.18±0.02	0.6±0.02	6.5±1.33	>50	3.8±0.61
9	AA105	<$>\scale 75%\raster="rg9"<$>	855.2	3±0.2	0.9±0.14	2.7±0.13	16.1±0.78	>50	>50

### Antibodies

The antibodies used in this study were as follows: anti-β-Actin (Sigma); anti-p-AMPKα1, anti-p-ACC, anti-p-mTOR, anti-p-S6K, anti-AMPKα1, anti-ACC, anti-mTOR, anti-S6K, anti-PARP, goat anti-rabbit IgG-HRP and goat anti-mouse IgG-HRP antibody (Cell Signaling Technology); anti-CyclinD1 (Abcam), anti-CDK4 (Santa Cruz Biotechnology), and anti-LC3 (Sigma).

### Cell Culture and Transfection

The lung cancer cell line A549, breast cancer cell line MCF-7, cervical cancer cell line HeLa, colon cancer cell lines LOVO, SW480, HCT116 and HT29, and human embryonic kidney HEK-293T cells were obtained from the American Tissue Culture Collection (ATCC). Human embryonic lung fibroblast MRC-5, gastric cancer cell line SGC7901, and hepatic cancer cell line BEL7402 were purchased from the Cell Resource Center, Chinese Academy of Medical Sciences (Beijing). The normal human bronchial epithelial cells (HBEpiC) were purchased from ScienCell (ScienCell Research Laboratories, San Diego, California). The 293T, A549, BEL7402, HeLa, MCF-7, SGC7901 and the human normal bronchial epithelial cells BEAS-2B [29] cells were cultured in Dulbecco modified Eagle medium (DMEM) containing 10% fetal bovine serum (FBS; Gibco/BRL, Grand Island, NY), 100 U/ml penicillin and 100 µg/ml streptomycin. LOVO, SW480, HCT116 and HT29 cells were cultured in DMEM/F12 supplemented with 10% FBS, 100 U/ml penicillin and 100 µg/ml streptomycin. MRC-5 cells were cultured in MEM/EBSS medium supplemented with non-essential amino acids, 10% FBS, 100 U/ml penicillin and 100 µg/ml streptomycin. HBEpiC cells were cultured in a serum-free bronchial epithelial cell medium (ScienCell Research Laboratories) containing bronchial epithelial cell growth supplement (ScienCell Research Laboratories). The pQCXIP-GFP-LC3 and pQCXIP-GFP plasmids [30] were transfected into LOVO cells using the Lipofectamine 2000 (Qiagen) according to the recommended protocol by the manufacturer.

### Cell Viability, Cell Proliferation and Clonogenic Assays

Cancer cells (5×10^3^) were seeded in each well of 96-well tissue culture plates (Coaster, Charlotte, NC) and treated with annonaceous acetogenin mimetics for 48 h at 37°C in a 5% CO_2_ atmosphere. MTT assay was performed as described [31], and the IC_50_ values were calculated using the CalcuSyn software (version 2.0, Biosoft, Cambridge, UK). Cell viability was estimated by trypan blue dye exclusion. The potential synergistic, additive or antagonistic effect between AA005 and 2-DG or cisplatin was carefully assessed using the Calcusyn Software (Biosoft, Cambridge, UK) as described [32]. The dose-effect curves of single or combined drug treatment were analyzed by the median-effect method [33], where the combination indexes (CI) less than, equal to, and greater than 1 indicate synergistic, additive, and antagonistic effects, respectively.

For foci formation, HT29, LOVO or HCT116 treated with AA005 were seeded in triplicate into 35 mm plates (200 cells per plate). After 8 days of culturing, cells were stained with Giemsa and clones containing more than 50 cells were counted [29].

### Analysis of Cell Cycle and Apoptosis

To detect the cell cycle distribution, colon cancer cells were synchronized to G1/S boundary by a double thymidine block [31] and then exposed to AA005 at indicated concentrations for 24 h. Cells were harvested, fixed with 70% cold ethanol in 4°C overnight. The cells were centrifuged and washed with PBS, followed by incubation with RNase and propidium iodide (PI) (Sigma-Aldrich). Cell cycle distribution was analyzed by flow cytometry (BD FACS Vantage Diva, USA). Cell apoptosis was measured using PE Annexin V/PI Apoptosis Detection kit (BD Biosciences, San Jose, CA) according to manufacturer’s instruction.

### Analysis of Cells with GFP-LC3 Vesicles

Cells were transfected with pQCX-IP-GFP-LC3 and pQCX-IP-GFP-expressing plasmids for 24 h, and then treated with various concentrations of AA005 for another 24 h. Cells were fixed with 4% paraformaldehyde/PBS for 15 min at room temperature and analyzed using confocal microscopy at 63× magnification. The percentage of GFP-positive vesicles cells was assessed [30].

### Measurement of Mitochondrial Transmembrane Potential, ATP Content and NAD^+^/NADH Ratio

Cells were treated with different concentrations of annonaceous acetogenin mimetics for 24 h. The mitochondrial transmembrane potential was examined as described [34], ATP content was measured using an ATP Bioluminescence Assay Kit (Beyotime Institute of Biotechnology), and NAD^+^/NADH ratio was measured using Amplite™ Colorimetric NAD/NADH Assay Kit (AAT Bioquest, Inc., Sunnyvale, CA) according to the manufacturer’s instructions.

### Confocal Microscopy Analyses

Cells were grown on coverslips and fixed in 4% paraformaldehyde. After a brief washing in PBS supplemented with 100 mM glycine, slides were blocked with 5% bovine serum albumin (BSA; Sigma) and 0.3% Triton X-100 in PBS for 30 min at room temperature, and stained for fluorescence microscopy as described [31]. Cells upon AA005-flu were examined by use of a Zeiss LSM 510 META microscope equipped with a 63× oil-immersion objective. Image processing and analysis were done with Zeiss LSM 510 software version 3.2, ImageJ Version 1.42 (National Institutes of Health), and Adobe Photoshop Version 7.0 (Adobe Systems).

### Western Blotting

Cell pellets were lysed in RIPA buffer containing 50 mM Tris-HCl pH 7.4, 150 mM NaCl, 0.1% SDS, 1% deoxycholate, 1% TritonX-100, 1 mM EDTA, 5 mM NaF, 1 mM sodium vanadate, and protease inhibitors cocktail (Sigma). Cells were lysed on ice for 30 min in RIPA buffer, lysates were centrifuged, protein extracts were quantitated and loaded on 10% to 15% sodium dodecyl sulfate polyacrylamide gel, electrophoresed, and transferred to a nitrocellulose membrane (Whatman). The membrane was incubated with primary antibody, washed, and incubated with horseradish peroxidase (HRP)–conjugated secondary antibody. Detection was performed by using a chemiluminescent western detection kit (Cell Signaling Technology) [35].

### Statistical Analysis

All experiments were repeated at least three times and the data are presented as the mean±SD unless noted otherwise. Differences between data groups were evaluated for significance using Student *t*-test of unpaired data or one-way analysis of variance and Bonferroni post-test. *P* values<0.05 were considered statistically significant.

## Results

### Structure activity Relationship (SAR) Analysis of Annonaceous Acetogenin Mimetics

A serial annonaceous acetogenin mimetics had been synthesized by replacement of both tetrahydrofuran (THF) rings of natural bullatacin with a simple diethylene glycol ether unit by the chemistry group of our team [25]–[28]. Nine novel analogs were synthesized in this work, and we found that the mimetics AA005 [25] and AA093, a compound with the introduction of a 10-hydroxy group onto AA005, represented the two compounds which had the lowest IC50 values in this setting (Table 1). The observation that compound AA090 and AA091 lacking the right lactone unit and left 11-carbon tail exhibited a 17 to >170 fold lower cytotoxicity in cancer cells suggested that these groups are essential for the cytotoxicity of AA005. AA101 with an additional lactone unit embedded in the left hydrocarbon chain part exhibited a 23–142 fold lower cytotoxicity in cancer cells, further confirming the importance of the long hydrophobic tail and the right terminal lactone in the mimicry. Adding a middle ether unit to the mimetics (compounds AA102-105) slightly enhanced their anti-proliferative activity as compared to AA101, suggesting that a diethylene glycol ether unit is essential for the anti-proliferative activity. The diverse biological activity of these mimetics indicates that the structural analogs may not be functional analogs.

### Inhibitory Effects of AA005 on Cancer Cells

**Figure 1:**
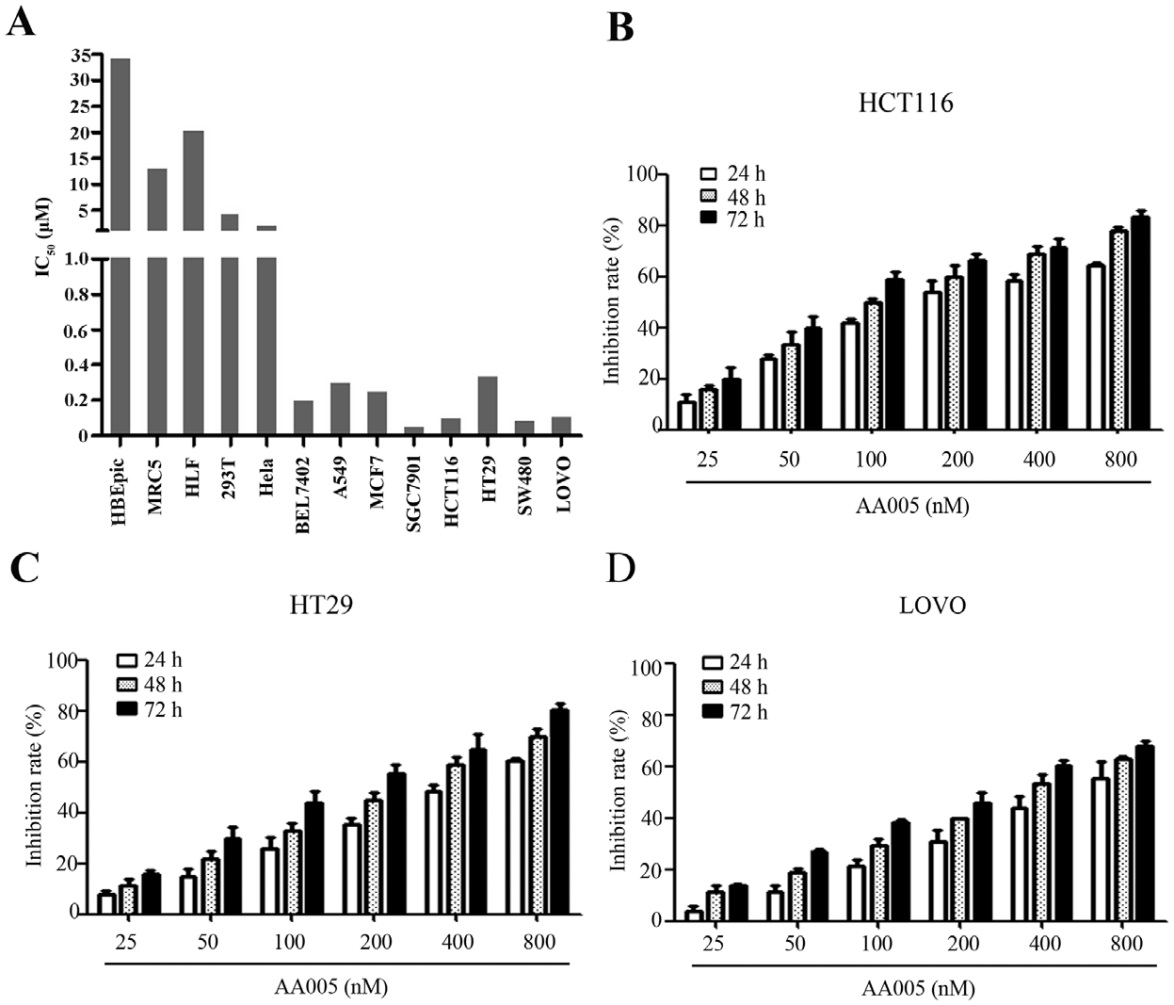
AA005 shows a relatively selective cytotoxicity against cancer cells. (A) IC_50_ values of AA005 (in 48 h) for various human cancer and noncancerous cell lines. IC_50_ values (mean ± SD, µM) were calculated from 3 independent experiments. (B through D) MTT assays of HCT116 (B), HT29 (C) and LOVO (D) cells upon AA005 at indicated concentration and time points.

Because AA005 was the most potent cytotoxic agent among these mimetics, we further tested its effects on 11 human cancer cell lines and 4 noncancerous cell lines (HBEpiC, MRC5, HLF and 293T), and found that AA005 showed diverse effects on cancer cells in that it had potent inhibitory effect on colon (HCT116, HT29, LOVO and SW480), gastric (SGC7901), hepatic (BEL7402), lung (A549) and breast (MCF7) cancer lines, and weak effect on cervical (HeLa) cancer cells (Figure 1A). AA005 exhibited inhibitory effects on HCT116 (Figure 1B), HT29 (Figure 1C) and LOVO (Figure 1D) cells in a dose- and time-dependent fashion. Interestingly, AA005 showed an even weaker activity against noncancerous (HBEpiC, MRC5, HLF, BEAS-2B and 293T) cells (Figure 1A and Table 1). These results indicate that the relative selective inhibitory effects of AA005 on cancer cells warrant further investigation.

### AA005 Suppresses Cell Proliferation and Colony Forming Activity of Colon Cancer Cells

**Figure 2:**
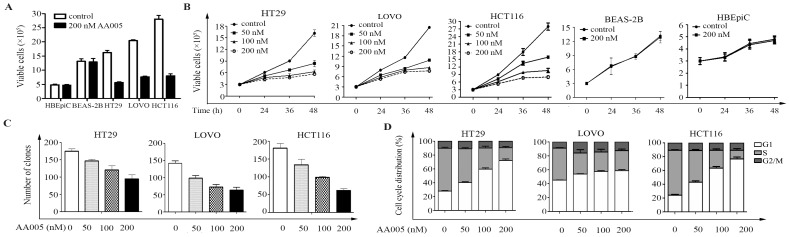
AA005 inhibits cell growth/proliferation, suppresses colony forming activity and arrests cell cycle in colon cancer cells. (A, B) Indicated cells were treated with or without AA005 for 48 h or indicated time points, and analyzed by trypan blue exclusion assay. (C) Colony formation assay for the clonogenic activity of colon cancer cells treated with or without AA005. (D) Colon cancer cells were treated with AA005 at indicated concentrations for 24 h. Cell cycle distribution was determined by flow cytometry.

We further analyzed the effects of AA005 on colon cancer cells. By using the trypan blue exclusion analyses, we showed that treatment with AA005 at 50 to 200 nM for 24 to 48 h markedly inhibited proliferation of HT29, LOVO and HCT116, but not HBEpiC or BEAS-2B cells (Figure 2, A and B). Foci formation assay showed AA005’s potent inhibitory effects on colony forming activity of colon cancer cells (Figure 2C). We analyzed the effects of AA005 on cell cycle and found that AA005 caused a substantial increase in the percentage of colon cancer cells in G1 phase in a dose-dependent fashion (Figure 2D).

### AA005 Targets Mitochondria, Depletes ATP and Activates AMPK in Colon Cancer Cells

**Figure 3:**
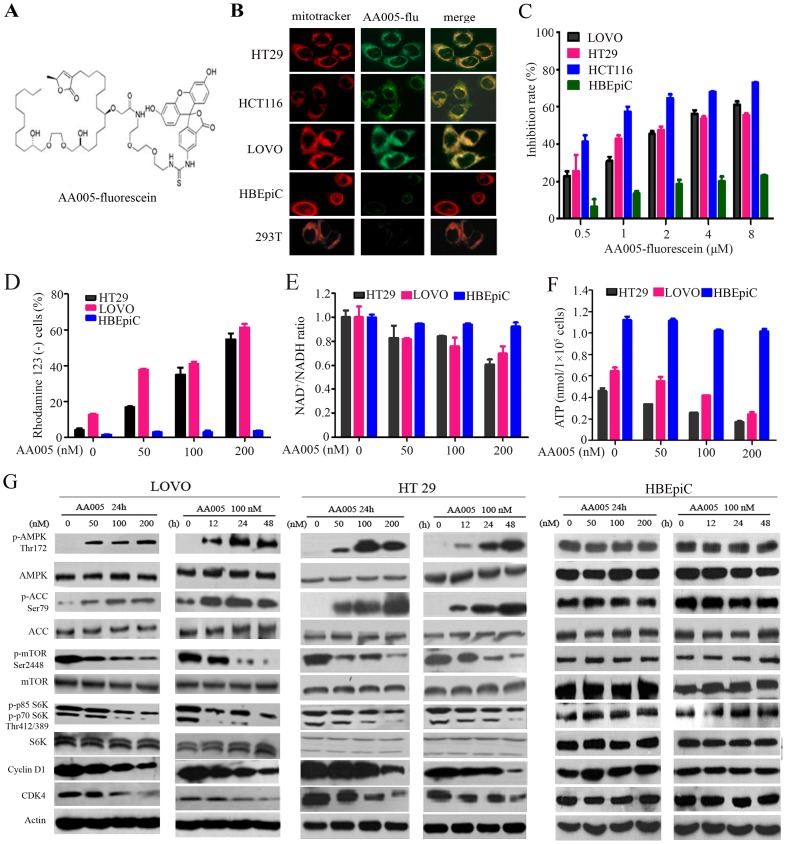
Effects of AA005 on ATP production and AMPK/mTOR signaling pathway. (A) Chemical structure of AA005-fluorescein. (B) The intracellular localization of AA005. The cells were co-incubated with AA005-flu at 100 nM for 12 h, and analyzed by confocal microscopy using a mitotracker (red) to counter-stain mitochondria. (C) MTT assay of LOVO, HT29, HCT116 and HBEpiC cells upon AA005-flu at indicated concentrations for 48 h. (D) AA005 decreases the mitochondrial transmembrane potential of colon cancer cells revealed by increase in Rhodamine 123-negative cells. The cells were treated with AA005 at indicated concentration for 24 h and analyzed by Rhodamine 123 staining and flow cytometry. (E) The cells were treated with or without AA005 at indicated concentration for 24 h, NAD^+^/NADH ratio was measured using an AmpliteTM Colorimetric NAD/NADH Assay Kit. (F) The cells were treated with or without AA005 at indicated concentration for 24 h, and ATP content was measured using an ATP Bioluminescence Assay Kit. (G) The cells were treated with AA005 at indicated concentration and time points, lysed, and Western blot analysis was performed using indicated antibodies.

Fluorescein-labeled AA005 (AA005-flu, Figure 3A) was successfully accomplished by a biological activity assessment-aided protocol after examining a number of potential derivative positions in parallel [36]. AA005-flu was found to exhibit similar cell selectivity to its parental molecule, and accumulate in the mitochondria of hepatic cancer but not normal cells [36]. By using immunofluoresence confocal microscopy analysis, we demonstrated that AA005-flu could co-localize with mitochondria in HT29, HCT116 and LOVO cells (Figure 3B). However, AA005-flu signal was very weak in mitochondria of HBEpiC or 293T cells (Figure 3B). While AA005-flu inhibited proliferation of LOVO, HT29 and HCT116 cells in a dose-dependent fashion, its cytotoxic effect on HBEpiC was weak (Figure 3C). Furthermore, we reported that AA005 could increase Rhodamine 123-negative fractions in HT29 and LOVO but not HBEpiC cells (Figure 3D). These results suggest that AA005 might target mitochondrial molecules and thus perturb energetic pathway of cancer cells.

Mitochondria targeting agents (mitocans) are prone to disrupting oxidation phosphorylation pathway and reducing NAD^+^/NADH ratio, resulting in inhibition of ATP production and activation of AMPK that is reflected by phosphorylation and inactivation of Acetyl-CoA Carboxylase (ACC) (Ser79) which is an indicator for AMPK activity [37]. We tested the effect of AA005 on cellular NAD^+^/NADH ratio and ATP content, and found that in HT29 and LOVO cells, treatment with AA005 at 50 to 200 nM for 24 h reduced NAD^+^/NADH ratio (Figure 3E) and depleted ATP in a dose-dependent manner (Figure 3F), while interestingly, AA005 could not significantly decrease NAD^+^/NADH ratio and ATP content in HBEpiC cells (Figure 3E and F). Furthermore, AA005 markedly up-regulated p-AMPK and p-ACC in a dose- and time-dependent fashion (Figure 3G). However, these phenomena were not observed in HBEpiC cells (Figure 3G).

**Figure 4:**
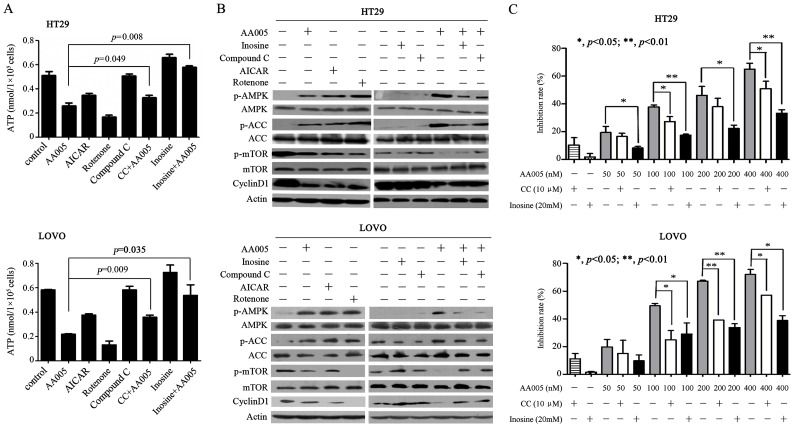
AMPK activation is required for the effects of AA005 on colon cancer cells. (A) The indicated cells were treated alone or combinatory with AA005 (100 nM), AICAR (AA, 1 mM), retenone (1 µM), compound C (CC, 10 µM), or inosine (20 mM) for 24 h, and ATP content was measured. (B) HT29 and LOVO cells were treated with indicated protocol, lysed, and Western blotting was conducted using indicated antibodies. (C) HT29 and LOVO cells were treated with different treatment regimens for 48 h, and cell viability was detected by MTT assay.

We showed that AA005 as well as the mitocans AICAR and rotenone caused ATP depletion in HT29 and LOVO cells, while AMPK inhibitors compound C and inosine attenuated this effect (Figure 4A). AA005, AICAR and rotenone up-regulated p-AMPK and p-ACC, while compound C and inosine reduced AA005-triggered up-regulation of the two molecules (Figure 4B). In addition, while AA005 inhibited proliferation of HT29 and LOVO cells, compound C and inosine reduced this effect (Figure 4C). These results suggest that AMPK activation is required for AA005-induced cytotoxicity to colon cancer cells.

### AMPK Activation Mediates AA005-induced mTOR Complex 1 (mTORC1) Inhibition in vitro

Activated AMPK phosphorylates TSC2 at Thr-1227 and Ser-1345 and increases the activity of TSC1–TSC2 complex to inhibit mTOR [12]. We tested the effects of AA005 on mTORC1, and reported that AA005 decreased p-mTOR and p-S6K (p85 and p70 S6K) in LOVO and HT29 but not HBEpiC cells (Figure 3G). AICAR and rotenone also down-regulated p-mTOR in HT29 and LOVO cells (Figure 4B). However, AA005-induced p-mTOR down-regulation could be partially repressed by inosine and compound C (Figure 4B). Compound C and inosine also partially attenuated cyclin D1 down-regulation caused by AA005 (Figure 4B).

### AMPK/mTOR Signaling is Involved in AA005 Induced Autophagy in vitro

**Figure 5:**
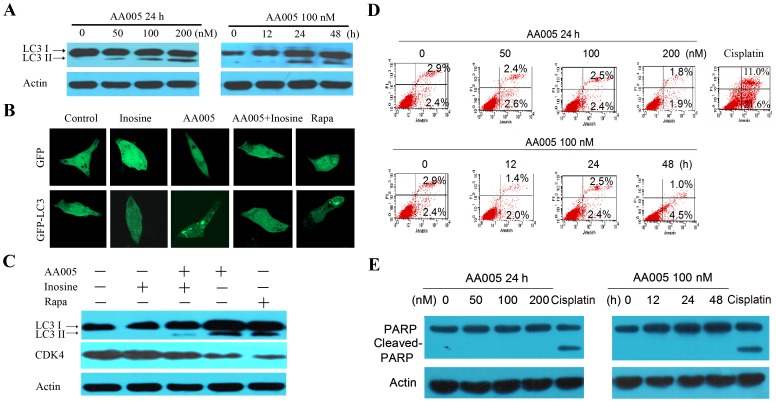
AA005 induces autophagy of colon cancer cells. (A) Immunoblot analysis of LC3-I and LC3-II levels in LOVO cells treated with AA005. (B) LOVO cells transfected with pQCXIP-GFP or pQCXIP-GFP-LC3 plasmid were treated for 24 h with AA005 (100 nM) and/or inosine (20 mM), and rapamycin (100 nM), and assessed by immunofluorescence analyses. (C) LOVO cells were treated with indicated protocols, lysed, and Western blot assay was performed using indicated antibodies. (D) LOVO cells were treated with AA005 or cisplatin (20 µM) for 24 h, or AA005 at 100 nM for indicated time points. The cells were then analyzed by Annexin V/PI staining and flow cytometry. (E) Western blot analyses of lysates of LOVO cells treated with AA005 or cisplatin (20 µM for 24 h).

AMPK activation can induce autophagy via inhibition of mTOR (49). We tested whether AA005 could induce autophagy in colon cancer cells or not by detecting the changes of cytosolic form (LC3-I) and lipidated form (LC3-II) of the autophagy marker LC3. Interestingly, we found that AA005 induced accumulation of LC3-II in LOVO cells in a time- and dose-dependent fashion (Figure 5A). The pQCXIP-GFP-LC3 plasmid was transfected into LOVO cells which were then treated with AA005 at 100 nM for 24 h, followed by confocal microscopy assessment. We showed that while control cells displayed a diffuse staining, LOVO cells upon AA005 or mTOR inhibitor rapamycin (100 nM) exhibited a speckled fluorescent staining pattern, indicating the redistribution of LC3 to autophagosomes (Figure 5B). Interestingly, inosine attenuated AA005-caused formation of LC3 autophagosomes (Figure 5B) and accumulation of LC3-II (Figure 5C). These results indicate that AA005 induces autophagy of colon cancer cells via AMPK/mTOR signaling pathway. However, treatment with AA005 at 50–200 nM for 24 h or 100 nM for 12–48 h did not result in marked apoptosis of LOVO cells, reflected by Annexin V/PI staining and flow cytometry assessment (Figure 5D) or Western blot analysis of cleavage of PARP, a substrate of activated casp-3 (Figure 5E).

### AA005 Synergizes with 2-Deoxyglucose and Cisplatin in Inhibiting Colon Cancer Cell Proliferation by Modification of AMPK and mTOR

**Figure 6:**
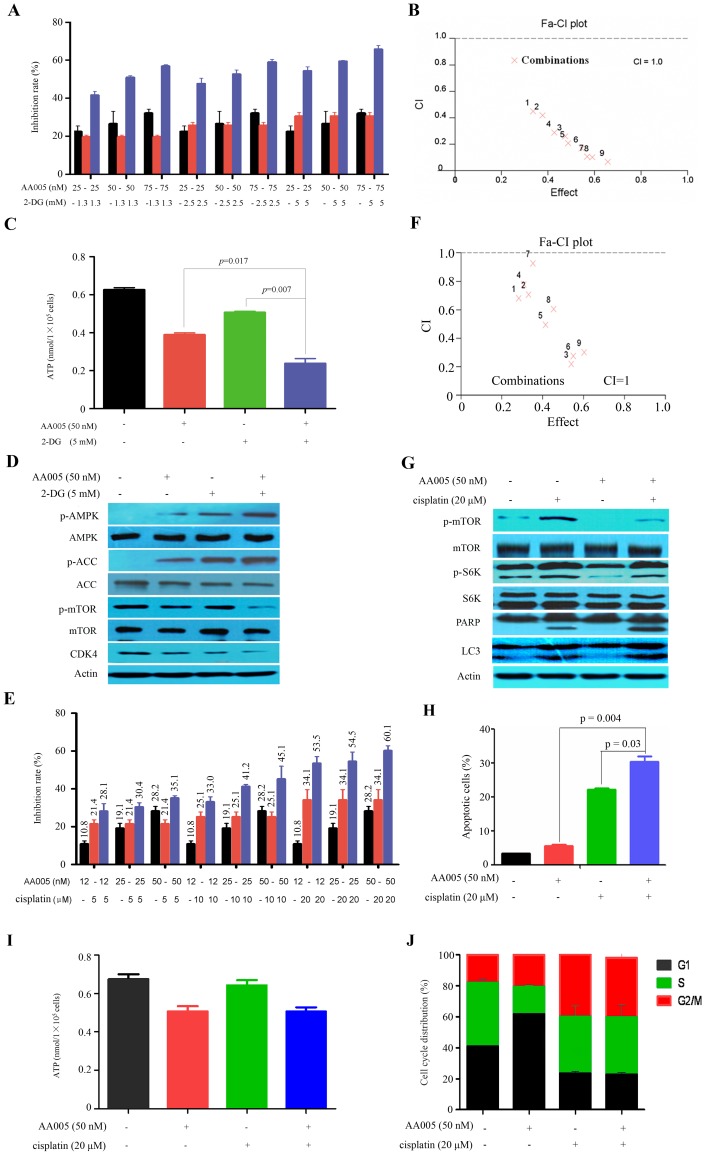
AA005 synergizes with 2-DG and cisplatin in colon cancer cells. (A, B) LOVO cells were treated indicated protocols for 48 h, analyzed by MTT assay (A), and the combined effects were evaluated by the Chou-Talay method and Calcusyn software (B). The combination indexes (CI) less than, equal to, and greater than 1 indicate synergistic, additive, and antagonistic effects, respectively. (C) LOVO cells were treated with 50 nM AA005 or/and 5 mM 2-DG for 24 h, and ATP content was assessed as described above. (D) Western blot analyses of lysates of LOVO cells treated with AA005 or/and 2-DG using indicated antibodies. (E, F) LOVO cells were treated indicated protocols for 48 h, analyzed by MTT assay (E), and the combined effects were evaluated by the Chou-Talay method and Calcusyn software (F). (G) Western blot analyses of lysates of LOVO cells treated with AA005 or/and cisplatin using indicated antibodies. (H) LOVO cells were treated with AA005 and/or cisplatin, and analyzed by Annexin V/PI staining and flow cytometry. (I) The cells were treated with AA005 and/or cisplatin for 24 h, and ATP content was measured using an ATP Bioluminescence Assay Kit. (J) LOVO cells were treated with AA005 and/or cisplatin for 24 h, and cell cycle distribution was determined by flow cytometry.

2-Deoxyglucose (2-DG) is a synthetic glucose analogue capable of inhibiting glycolysis and ATP production [38]. We tested the combined effects of AA005 and 2-DG in LOVO cells, and found that combined use of the two agents exerted synergism in inhibition of cell proliferation (Figure 6A and B) and suppression of ATP generation (Figure 6C). AA005 in combination with 2-DG led to enhanced up-regulation of p-AMPK and p-ACC, and down-regulation of p-mTOR and CDK4 (Figure 6D).

Chemotherapeutic agent cisplatin can up-regulate mTOR survival pathway which confers drug resistance to cancer cells [39]. We showed that while AA005 and cisplatin caused synergistic inhibitory effect on LOVO cell proliferation (Figure 6E and F), AA005 decreased cisplatin-triggered up-regulation of mTOR and S6K (Figure 6G). Combined use of AA005 and cisplatin led to enhanced apoptotic effect on LOVO cells, reflected by Annexin V/PI staining and flow cytometry assessment (Figure 6H) or Western blot analysis of cleavage of PARP (Figure 6G). However, combination of these two agents did not cause synergy in depletion of ATP content (Figure 6I), cell cycle arrest (Figure 6J), or autophagy (reflected by the expression LC3-II, Figure 6G) in LOVO cells.

## Discussion

Annonaceous acetogenins represent a series of C-35/C-37 natural products with hydroxylated THF and the γ-lactone rings structures [25], [26], [28]. Many members of this family display cytotoxic activity by perturbation of the terminal electron transfer step in complex I of mitochondria [40]. AA005 is a mimetic of annonaceous acetogenin in which both the THF rings are replaced by an ethylene glycol ether unit. AA005 retains the essential functionalities of the natural acetogenins and shows more powerful biological activity [41]. In this study, we report that AA005 is able to activate AMPK and inhibit mTOR, therefore arrests cell cycle at G1 phase and induces autophagy of colon cancer cells. AA005 synergies with glycolysis inhibitor 2-DG for marked ATP generation blockade, and antagonizes cisplatin-induced up-regulation of p-mTOR, leading to enhanced proliferation inhibition and apoptosis induction in colon cancer cells. These results indicate that AA005 bears therapeutic potentials at least in this kind of malignant neoplasm.

Targeting cancer cell metabolism, especially glycolysis inhibition, has emerged as a new promising strategy to fight cancer [42]–[44]. Mitochondria not only manage energy generation via citric acid cycle (tricarboxylic acid cycle, TCA cycle), but also play a key role in apoptosis regulation through release of cytochrome C. Although there has much debate on whether mitochondria have defects in oxidative phosphorylation pathway, it could be a potential target for cancer therapy [42], [45]. Some mitocans such as metaformin and vitamin E analogues which inhibit complex I and II show effective and selective anti-cancer activity. These agents induce cancer cell death through generation of superoxide and mitochondrial destabilization [46]. ATP could also be depleted to some extent upon treatment of mitocans [42], [45]. Mitochondria complex I has been shown to be targeted by annonaceous acetogenin [40]. We report that AA005 can co-localize with mitochondria in colon cancer but not HBEpiC or 293T cells (Figure 3A), suggesting that AA005 may reduce NAD^+^/NADH ratio and ATP production in cancer cells. This possibility is confirmed in LOVO, HT29 and HCT116 cells (Figure 3C). However, why mitochondria in cancer cells are more prone to be targeted by AA005 remains an open question. Inhibition of mitochondria by metaformin and rotenone may lead to activation of AMPK [42]. We show that AA005 can also suppress mitochondria and activate AMPK (Figure 3). At this point, inosine and compound C antagonize AA005-induced AMPK activation and cytotoxicity (Figure 4), indicating a direct link between ATP depletion through mitochondria inhibition and AMPK activation, which ultimately results in mTOR inhibition. AA005 also synergizes with 2-DG in ATP depletion and AMPK activation, indicating that AA005 bears therapeutic potentials for colon cancer.

The oncoprotein mTOR suppresses while activation of AMPK promotes autophagy [47]. We show that AA005 down-regulates p-mTOR as well as its downstream effector S6K (Figure 3), while compound C and inosine partially reverse this effect (Figure 4). Inosine also represses AA005-induced autophagy of LOVO cells reflected by decrease LC3-II accumulation (Figure 5C). While cisplatin up-regulates mTOR, AA005 attenuates this phenomenon (Figure 6G) and synergizes with this agent in inducing apoptosis of LOVO cells (Figure 6H). These results indicate that AA005 can inhibit mTOR via activation of AMPK, and further demonstrate the benefits of this annonaceous acetogenin mimetic for colon cancer cells.
